# The double-edged sword effects of leader perfectionism on employees' job performance: the moderating role of self-efficacy

**DOI:** 10.3389/fpsyg.2025.1412064

**Published:** 2025-01-28

**Authors:** Lingjiang Zhao, Haishen Huang

**Affiliations:** School of Business Administration, South China University of Technology, Guangzhou, China

**Keywords:** leader perfectionism, harmonious work passion, performance pressure, job performance, self-efficacy, appraisal theory of emotion

## Abstract

The pursuit of perfection has become a common job requirement for modern organizations against the backdrop of increasingly fierce market competition. Drawing upon appraisal theory of emotion, we develop and test a model delineating the paradoxical role that leader perfectionism plays in predicting employee's job performance. Specifically, we propose that leader perfectionism improves job performance through increased harmonious work passion and simultaneously hinders it through enhanced performance pressure. We further propose self-efficacy as boundary condition, such that the positive path through harmonious work passion is stronger and the negative path through performance pressure is weaker for individuals with higher (vs. lower) levels of self-efficacy. Finding from a three-wave and multisource field study provides support for our hypothesized predictions. Finally, we discuss theoretical and practical implications of this study.

## 1 Introduction

The pursuit of perfection is pervasive across society (Sirois and Molnar, 2016). In business circles, the renowned Japanese entrepreneur Kazuo Inamori has seen perfectionism as one of the key principles of his management philosophy (Kase et al., 2022), positing that only if striving for perfection can business be excellently met with success. In academia, some management scholars argue that “perfection should influence our day-to-day and perhaps even moment-to-moment activities” (Baer and Shaw, 2017, p. 1215). Perfectionism is defined as an individual's intrinsic trait of seeking or demanding ideal or extremely high performance standards from oneself or others (Frost et al., 1990; Ocampo et al., 2020; Shoss et al., 2015). Due to the prevalence of perfectionism in the workplace, in recent years, organizational behavior scholars have gradually turned their research focus to perfectionism and promoted the research in this field (Ocampo et al., 2020, 2024; Lin et al., 2023; Jiang F. et al., 2024; Wang et al., 2024). Most of the existing studies have investigated the maladaptive results of perfectionism (Harari et al., 2018), showed that perfectionism's high focus on uncertainty and error increases the sense of control to others and requests others to behave in the same way as a perfectionist, thus inhibiting followers' positive behavior and performance (Ozbilir et al., 2015; Rice and Liu, 2020; Kinman and Grant, 2022; Cîrsmari et al., 2023; Xiong and Zhang, 2023).

However, despite the negative implications of perfectionism, recent research has suggested that this trait may also have a bright side. Specifically, leader perfectionism may enhanced job conscientiousness (Stoeber et al., 2009) and work engagement (Childs and Stoeber, 2010), which imply that leader perfectionism may promote potential positive outcomes, such as employees' job performance. Considering these paradoxical findings, one possible explanation for this contradiction is that perfectionism's negative effect may overshadows its positive effect (Harari et al., 2018), and we should analyze relationships through multiple different mediators (Xu et al., 2022; Jiang X. et al., 2024). Recent studies found that perfectionism can influence job performance by affecting individual goal-setting (Hrabluik et al., 2012), job demand-resource (Gunyakti Akdeniz et al., 2024), or leader-member relationship (Song et al., 2022). These findings provide a partial picture of the relationship between leader perfectionism and job performance. Although employees may feel the pressure from prefect performance command, they may also be engaged in work proactively to narrow the gap between the current and desired states (Harju et al., 2021). Therefore, the intermediate mechanisms and boundary conditions underlying the influences of leader perfectionism on followers' performance were not fully addressed.

Drawing on appraisal theory of emotion, we propose that leader perfectionism leads to two distinct emotional appraisals for employees: harmonious work passion and performance pressure. *Harmonious work passion* refers to a emotional state where an individual, driven by intrinsic motivation, invests time and effort in beloved work activities (Robertson and Barling, 2013; Vallerand et al., 2003, 2007). *Performance pressure* refers to a negative emotional response that current performance is insufficient to meet performance goal (Eisenberger and Aselage, 2009; Kundi et al., 2022). On the one hand, perfectionistic leaders show conscientiousness toward subordinates (Stoeber et al., 2009), concentrate their passion and effort into work, and set higher performance standards for their subordinates (Slade and Owens, 1998). Therefore, employees may imitate leader's behaviors and take the initiative to take efforts to work, triggering harmonious work passion. On the other hand, perfectionistic leaders often impose overly high-performance expectations and stringent evaluation standard on subordinates (Hill et al., 1997), which likely triggers performance pressure.^1^ Accordingly, leader perfectionism likely evokes both harmonious work passion and performance pressure in followers, which may result in different job performance.

Although the mechanism of positive and negative emotional appraisal may occur simultaneously, appraisal theory of emotion suggests that individual characteristics lead to differences in emotional appraisal, resulting in diverse emotional responses (Lazarus, 1991b). In light of the intense focus of leader perfectionism on job performance, we examine an employee characteristic that causes them to respond differently to the ever-increasing performance standards of leader perfectionism. Therefore, we look to employee self-efficacy as moderator, defined as a belief about their capabilities to produce designated levels of performance (Bandura, 1982). It is theoretically relevant here because employees believe in their capacity to meet high-performance standards when they have higher self-efficacy (Chen et al., 2016), which further magnifies the positive role of leader perfectionism. Conversely, lower self-efficacy in employees may amplify the negative impact of leader perfectionism due to increased concern over performance expectations.

Our research contributes to the scholarship on leader perfectionism and related research in three ways. First, we paint a more comprehensive picture of the consequences of leader perfectionism. In spite of previous research has reflected the negative social function of perfectionism on others (Curran and Hill, 2017; Lin et al., 2023), there may be unintended positive outcomes for perfectionistic leaders in the workplace (e.g., Song et al., 2022; Xu et al., 2022). Therefore, we focus on the emotional appraisal perspective to reveal the complexity of leader perfectionism by emphasizing the mixed effects of leader perfectionism on job performance. Second, by integrating literature of appraisal theory of emotion and perfectionism, we propose a model with dual paths that accounts for distinct emotional appraisal and contribute to perfectionism research. Thus, here we propose and test harmonious work passion and performance pressure as the link between leader perfectionism and employees' job performance. Our research provide a more balanced view of leader perfectionism that is a key underlying mechanism for the mixed effect of perfectionism and job performance (Harari et al., 2018). Third, we illustrate the role of self-efficacy in altering the impact of leader perfectionism, highlighting how individual characteristics can reconcile the consequences of leader perfectionism on employees' adaptive or maladaptive consequence.

## 2 Theory and research hypotheses

### 2.1 Leader perfectionism and emotional appraisal

Our research seeks to address this issue by providing a different theoretical perspective for understanding the paradoxical influences of leader perfectionism on employees' job performance. We draw upon appraisal theory of emotion as the guiding framework for our theoretical model. Appraisal theory of emotion posits that the evaluations and interpretations of events determine individuals' emotions (Roseman et al., 1990), resulting in different behaviors and performance (Choi et al., 2011). Emotions serve as the core of attitude formation and behaviors (Ashkanasy and Daus, 2002), the behaviors of leader “have an important influence on the moods and feelings of their followers” (Ashkanasy and Humphrey, 2011, p. 218). Hence, employees produce two distinct types of appraisal processes simultaneously and trigger positive or negative emotion (Lazarus, 1991a).

However, the complexity of perfectionism suggests the resulting emotional appraisal may be similarly complex. Individuals with perfection traits usually have high requirements for goals and extreme expectation toward others (Otto et al., 2021). According to appraisal theory of emotion, individuals may experience both positive and negative emotional appraisals to the same stimulus (Ellsworth, 2013; Schneider and Schwarz, 2017), especially when the stimulus has both positive and negative aspects (Larsen and McGraw, 2011). Such positive and negative elements may lead to ambivalence, as well as positive and negative emotions (Rothman et al., 2017). Hence, when confronted with the work attitudes and behaviors of perfectionistic leaders, employees may experience both positive and negative emotional appraisal and subsequently impact their job performance. In this research, we reason that employees may interpret these as the leaders' aspirations for them after perceiving their leaders' diligent work attitudes and high-performance expectations, thereby generating a positive emotional appraisal (i.e., harmonious work passion) (Gutermann et al., 2017) and negative appraisal (i.e., performance pressure).

### 2.2 The mediating role of harmonious work passion

Vallerand et al. (2003) were the first to introduce the concept of passion into the organizational research, describing work passion as the strong inclination or willingness of employees to love their work, fully identify with its value, and invest time and effort into it. Harmonious work passion stems from an individual's genuine love for their work and voluntary engagement, which shows that employees can control their own work.

According to appraisal theory of emotion, the emotional appraisal of external stressors is crucial in generating different emotional responses and subsequent behaviors (Moors et al., 2013). Emotion theorists posit that one person's emotion can lead to corresponding changes in another person's emotion by emotional appraisal to interpret emotion's meaning, finally generating specific discrete emotions (Parkinson, 2011). Extant research showed that leadership, as an important contextual factor, can promote employees' work passion (Egan et al., 2017, 2019). Specifically, leader, as the authority of the organization, followers will pay extra attention to his words and deeds and interpret his behaviors (Simons, 2002). The motivation of perfectionistic leader is a desire to finish the work with high quality, and it is an internal and heartfelt passion (Stoeber et al., 2013), which drives perfectionistic leaders to devote time and effort in their work spontaneously, such as working overtime and demanding details, to create a perfect-oriented team value (Palanski and Yammarino, 2011). In the workplace, subordinates generally regard the leader as a role model, and employees will consciously or unconsciously imitate the attitude or behavior of leader (Dietz et al., 2020). The emotion of leaders' devotion to work will be transmitted to subordinates (i.e., emotion contagion), and then increase employees' emotional appraisal of high work engagement (Parkinson, 2020), which leads to imitate leaders' time and effort to improve work and stimulate harmonious work passion (Li et al., 2017; Wo et al., 2019).

Existing research indicated that harmonious work passion, as a form of positive emotional state, facilitates job performance (Pollack et al., 2020). On the one hand, harmonious work passion can ignite strong work intentions in employees, leading to work engagement and effort toward achieving goals, thereby enhancing job performance (Vallerand et al., 2010; Zigarmi et al., 2018). On the other hand, employees with harmonious work passion, due to their focus and enthusiasm for work, are more likely to gain recognition and support from leaders, obtaining valuable resources such as training and promotion opportunities (Wang et al., 2022). The increase of harmonious work passion makes employees increase their work engagement and organizational identification, consequently improve their job performance (Burke et al., 2015; Astakhova and porter, 2015). Through effective integration and utilization of these resources, employees can not only complete work tasks with high quality but also engage in more organizational citizenship behaviors (McAllister et al., 2017; Shen et al., 2023) and employee voice (Gao and Jiang, 2019). As such, we hypothesize:

**H1:** Harmonious work passion mediates the relationship between leader perfectionism and job performance.

### 2.3 The mediating role of performance pressure

Performance pressure refers to the negative emotional state employees experience when they perceive their performance as insufficient to meet expected goals (Eisenberger and Aselage, 2009). Leader perfectionism often impose overly high-performance expectations and stringent evaluation standard on subordinates (Hill et al., 1997), and regard followers' performance as a part of their own performance, so they will pay great attention to subordinates' job performance (Guo et al., 2020). However, even if subordinates meet performance requirements, perfectionistic leaders may not experience satisfaction and set higher performance standards (Shafran et al., 2002). According to appraisal theory of emotion, because leader perfectionism has stringent performance standard, followers are likely to primarily appraise excessive performance demands as work pressure that thwarts their personal goals. For this reason, followers may generate passive emotional appraisal. For example, Hill and Curran (2016) found a positive correlation between a leader's perfectionism and employee job burnout, as employees facing extreme expectations might experience self-doubt and worry about their competency in their roles. Guo et al. (2020) also noted that leader perfectionism could negatively impact subordinates. On the one hand, employees might view performance pressure as an insurmountable barrier. Therefore, employees may regard performance pressure as an impossible barrier, leading to a significant positive impact of leader perfectionism on performance pressure.

Performance pressure generated by negative appraisal will bring a series of maladaptive consequences (Tan et al., 2021; Spoelma, 2022), because performance pressure make employees think that performance improvement is ineffective (Zhang et al., 2017), magnify the adverse effects of performance pressure, and thus inhibit their work performance. Kundi et al. (2022) discovered that performance pressure has varied effects on employee work engagement. Performance pressure perceived as a threat decreases work dedication, while pressure seen as a challenge increases it. Employee engagement has a significant positive impact on job performance, suggesting that challenging performance pressure can enhance job performance, whereas threatening performance pressure can undermine it. Mitchell et al. (2019) believe that performance pressure can be either a threat or a challenge, leading to positive or negative responses, meaning it can either motivate employees to perform well or lead to poor performance and inefficiency. The performance pressure felt by employees affects work efficiency, as it can foster motivation and functional work (Eisenberger and Aselage, 2009), thereby enhancing work efficiency. Xu et al. (2022) contend that leader perfectionism can have both positive and negative effects on employees. Based on this, we hypothesize:

**H2:** Performance pressure mediates the relationship between leader perfectionism and job performance.

### 2.4 The moderating role of self-efficacy

Self-efficacy refers to an individual's belief in their capability to execute specific tasks (Bandura, 1982). Appraisal theory of emotion indicates that individual trait differences lead to diverse emotional appraisals, resulting in distinct emotions (Lazarus, 1991a). Under environmental influences of leader perfectionism, employees with individual differences may tend to adopt different coping styles. Therefore, employees with high self-efficacy are more likely to trigger positive emotions and reduce the emergence of negative ones, leading to higher job performance, while those with low self-efficacy experience the opposite. Specifically, on the positive appraisal, employees with high self-efficacy, even when faced with the stringent work demands of perfectionistic leaders, believe they are capable of meeting these demands and make positive emotional appraisals, thereby generating more positive emotions, increasing harmonious work passion (Burić and Macuka, 2018). In contrast, employees with low self-efficacy perceive their abilities as insufficient to meet the endless pursuit of perfection by leaders, making it difficult to mobilize positive emotions to meet high performance standards. On the negative appraisal, employees with high self-efficacy are inclined to set higher work goals for themselves and continuously invest more time and effort (Lunenburg, 2011), internalizing the high performance standards set by perfectionistic leaders as their own work requirements (Bandura and Locke, 2003), thus feeling less performance pressure. Conversely, employees with low self-efficacy often set lower work goals, diverging from the high performance expectations of perfectionistic leaders (Slade and Owens, 1998). As a result, perfectionistic leaders are more likely to focus on these subordinates' performance and exert more work pressure (Guo et al., 2020), causing employees to feel increased performance pressure. As such, we hypothesize:

**H3a:** Employee self-efficacy positively moderates the relationship between leader perfectionism and harmonious work passion. The higher the employee's self-efficacy, the stronger the positive relationship between leader perfectionism and harmonious work passion.

**H3b:** Employee self-efficacy negatively moderates the relationship between leader perfectionism and performance pressure. The higher the employee's self-efficacy, the weaker the positive relationship between leader perfectionism and performance pressure.

Synthesizing the above discussion, we contend that employee self-efficacy moderates the indirect effects of leader perfectionism on job performance through harmonious work passion and performance pressure. Employees with high self-efficacy are more confident in meeting the high work demands from leaders and tend to experience more harmonious work passion when confronted with the leaders' pursuit of perfection, thereby increasing their work focus and improving job performance (Dubreuil et al., 2014). Conversely, employees with low self-efficacy fear that their abilities may not meet the leaders' extreme performance standards, and the substantial goal discrepancy between leaders and subordinates may lead to increased leader scrutiny of subordinate work performance, thereby heightening performance pressure and reducing job performance (Stetz et al., 2006). As such, we hypothesize:

**H4a:** Employee self-efficacy moderates the first stage of the positive indirect relationship between leader perfectionism and job performance through increased harmonious work passion, such that the relationship between leader perfectionism and harmonious work passion and, in turn, job performance is more positive when self-efficacy is higher rather than lower.

**H4b:** Employee self-efficacy moderates the first stage of the negative indirect relationship between leader perfectionism and job performance through increased performance pressure, such that the relationship between leader perfectionism and performance pressure and, in turn, job performance is less negative when self-efficacy is higher rather than lower.

**Figure 1:**
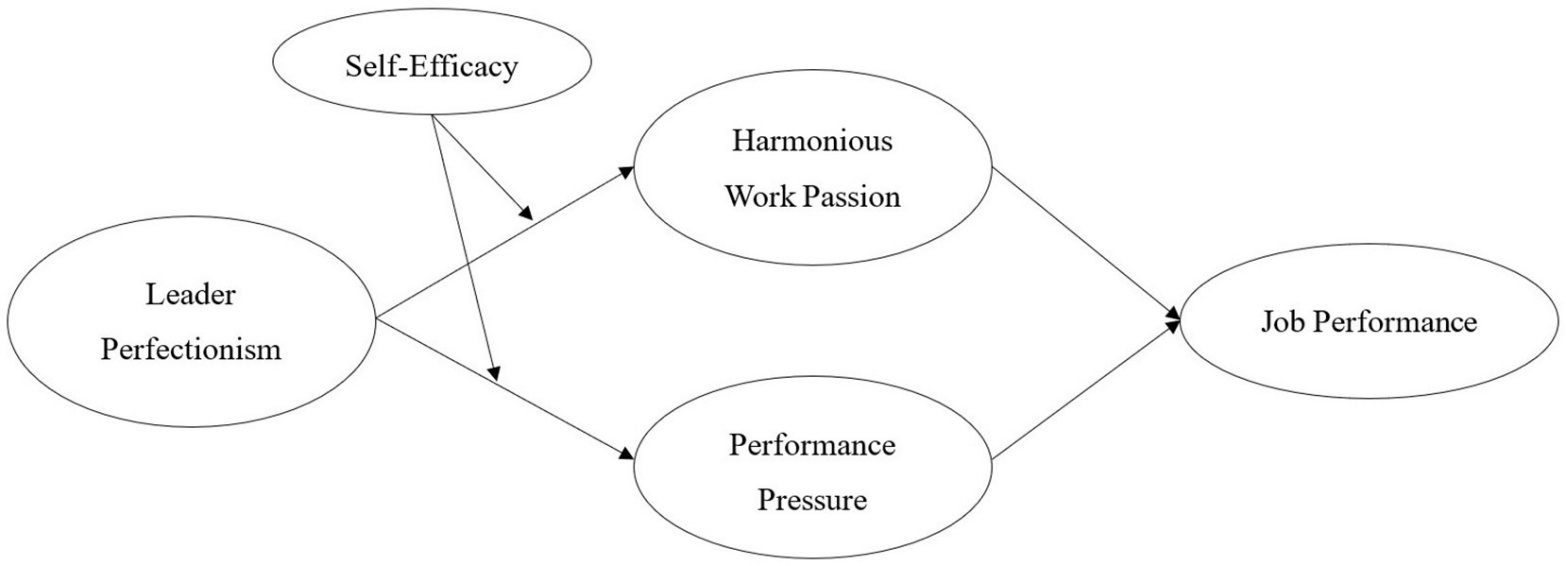
Theoretical model.

In summary, the theoretical model of this study is illustrated in Figure 1.

## 3 Method

### 3.1 Participants and procedures

To reduce the impact of common method bias, we conducted a multi-wave, multi-source field study with 1 month between waves. Before distributing the questionnaire, we selected the alumni who are managers and contacted 200 MBA alumni through the directory of a university in South China, assuring them of the research background, objectives, and procedures, and ensuring that data was for academic use only. After obtaining consent from the leaders, we sent out the survey questionnaires via email to the leaders and their subordinates, maintaining a record of questionnaire distribution and collection. In the end, a total of 89 leaders and 343 subordinates participated the survey.

At Time 1, employees were asked to rate their leader perfectionism and self-reported their self-efficacy and demographic information such as gender, age, and education. One month later (Time 2), we invited employees to rate their harmonious work passion and performance pressure. At Time 3 (1 month after Time 2), leaders evaluated job performance of their subordinates. Altogether, 277 completed employee surveys from 83 teams were used in the final analysis. an overall response rate of 80.8%, averaging 3.7 subordinates evaluated per leader. Of the 277 employees, 55.2% were male and 44.8% female. 23.1% under 25 years old, 30.7% between 26 and 30 years old, 24.9% between 31 and 35 years old, 14.1% between 36 and 40 years old, 5.4% between 41 and 45 years old, and 1.8% over 46 years old, with an average age of 30.8 years. 60.7% had a bachelor's degree or higher; and the average organizational tenure was 4.7 years.

### 3.2 Measures

We created Chinese versions for all measures following translation-back translation procedure (Brislin, 1970). All scales used a 5-point Likert scale, ranged from 1 (*strongly disagree*) to 5 (*strongly agree*).

#### 3.2.1 Leader perfectionism

A 5-item scale developed by Hewitt and Flett (1991) was used. A sample item was: “One of my leader goals is to be perfect in everything leader does.” The Cronbach's alpha was 0.80.

#### 3.2.2 Harmonious work passion

We used a 7-item scale developed by Vallerand et al. (2007) was used. A sample item was: “This activity reflects the qualities I like about myself.” The Cronbach's alpha was 0.82.

#### 3.2.3 Performance pressure

To measure performance pressure, we used (Tan et al., 2021) 6-item scale. A sample item was: “During work, I feel pressured to do a good job.” The Cronbach's alpha was 0.77.

#### 3.2.4 Self-efficacy

We adopted a 6-item scale developed by Maslach et al. (1997) was used. A sample item was: “I feel exhilarated when I accomplish something at work.” The Cronbach's alpha was 0.79.

#### 3.2.5 Job performance

We measured job performance with 5 items adapted from the measurement scale of Janssen and Van Yperen (2004). A sample item was: “This worker meets all the formal performance requirements of the job.” The Cronbach's alpha was 0.89.

#### 3.2.6 Control variables

We controlled for employees' gender, age, education, organizational tenure, and position level because previous studies showed they might influence the key variables (Ng and Feldman, 2008; LePine et al., 2016).

### 3.3 Analytic strategy

In our study, as each supervisor rated multiple employees' job performance, the data reflects a nested data structure. Considering that all variables were measured at individual level, we used design-based modeling to deal with the potential influence of non-independence (Wu and Kwok, 2012). Specifically, we set the command “TYPE = COMPLEX” and “ESTIMATOR = MLR” in Mplus 7.0 to use path analysis. Previous research has used this method to analyze similar data structure (Deng et al., 2023, 2024; Ren et al., 2023; Ni et al., 2024). To test the mediation and moderation effects, we followed recent studies (e.g., Lin et al., 2021) and used the Monte Carlo bootstrapping method with 20,000 replications to estimate the effects. The Monte Carlo approach can effectively explain the asymmetric distribution of indirect effects in single-level mediation context (MacKinnon et al., 2004; Preacher et al., 2010) and is recommended to test mediation effects (Selig and Preacher, 2008).

## 4 Results

### 4.1 Confirmatory factor analysis

To mitigate the impact of using the same data source on the statistical results, we used Harman's single-factor test to assess common method biases (Podsakoff et al., 2003, 2012). The results showed that the first factor explained 23.98% of the total variance, which falls below the threshold of 40%. Then, we conducted a confirmatory factor analysis on our focal variables. Because the ratio of our sample size to the estimated parameters is < 5:1, which does not meet the recommended ratio (Bentler and Chou, 1987), we used the random assignment for building parcels to improve the accuracy and validity (Little et al., 2002). Specifically, we parceled the items of harmonious work passion, performance pressure, and self-efficacy into three parcels each, while the other variables were not parceled. After parceling, the results as shown in Table 1, indicated that the five-factor model had the best fit compared to other models (χ^2^*/df* = 1.66, *CFI* = 0.96, *TLI* = 0.95, *RMSEA* = 0.05, *SRMR* = 0.04). Therefore, the common method biases in this study was not severe.

**Table 1:** Results of confirmatory factor analysis.

**Models**	***χ^2^***	***df***	***χ^2^/df***	**CFI**	**TLI**	**RMSEA**	**SRMR**
Five-factor model	235.47	142	1.66	0.96	0.95	0.05	0.04
Four-factor model	390.57	146	2.68	0.89	0.88	0.08	0.07
Three-factor model	463.54	149	3.11	0.87	0.85	0.09	0.07
Two-factor model	1,213.58	151	8.04	0.55	0.49	0.16	0.15
One-factor model	1,459.51	152	9.60	0.44	0.37	0.18	0.16

*N* = 277. Five-factor model: leader perfectionism; harmonious work passion; performance pressure; self-efficacy; job performance. Four-factor model: leader perfectionism; harmonious work passion + performance pressure; self-efficacy; job performance. Three-factor model: leader perfectionism; harmonious work passion + performance pressure + self-efficacy; job performance. Two-factor model: leader perfectionism; harmonious work passion + performance pressure + self-efficacy + job performance. One-factor model: all five variables were loaded on the same factor.

### 4.2 Reliability and validity

To measure the discriminate and convergent validity, we calculated the average variance extracted (AVE) value and composite reliability (CR). The results from Table 2 showed that AVE value was over 0.5, which was higher than Fornell and Larcker (1981)'s recommended value. The composite reliability (CR) value is higher than 0.7, indicating that the convergent validity among the variables meets the standard.

**Table 2:** Overall reliability and validity.

**Construct and indicators**	**AVE**	**CR**	**Loading**
*Leader perfectionism*	0.587	0.872	
1. It makes my leader uneasy to see an error in my work.			0.436
2. One of my leader's goals is to be perfect in everything his/her does.			0.872
3. My leader never aim for perfection in his/her work (R).			0.847
4. My leader must work to his/her full potential at all times.			0.770
5. My leader must always be successful at work.			0.821
*Harmonious work passion*	0.521	0.883	
1. This activity allows me to live a variety of experiences.			0.732
2. The new things that I discover with this activity allow me to appreciate it even more.			0.765
3. This activity allows me to live memorable experiences.			0.683
4. This activity reflects the qualities I like about myself.			0.760
5. This activity is in harmony with the other activities in my life.			0.577
6. For me it is a passion that I still manage to control.			0.825
7. I am completely taken with this activity.			0.686
*Performance pressure*	0.566	0.885	
1. During work, I feel pressured to do a good job.			0.805
2. During work, I feel I have to perform well.			0.797
3. During work, I feel pressured to produce results.			0.806
4. During work, I feel pushed to do well.			0.798
5. During work, I feel a lot of pressure to perform at a high level.			0.695
6. During work, I feel compelled to do the best I can.			0.585
*Self-efficacy*	0.535	0.873	
1. I feel exhilarated when I accomplish something at work.			0.640
2. I have accomplished many worthwhile things in this job.			0.747
3. I feel I'm making an effective contribution to what this Organization does.			0.768
4. I can effectively solve the problems that arise in my work.			0.812
5. In my opinion, I am good at my job.			0.641
6. At my work, I feel confident that I am effective at getting things done.			0.765
*Job performance*	0.710	0.924	
1. This worker always completes the duties specified in his/her job description.			0.880
2. This worker meets all the formal performance requirements of the job.			0.866
3. This worker fulfills all responsibilities required by his/her job.			0.858
4. This worker never neglects aspects of the job that he/she is obligated to perform.			0.756
5. This worker often fails to perform essential duties (R).			0.848

### 4.3 Descriptive statistics

The means, standard deviations, and correlation coefficients of the variables in this study are presented in Table 3. As expected, leader perfectionism was positively related to employees' harmonious work passion (*r* = 0.49, *p* < 0.01) and performance pressure (*r* = 0.35, *p* < 0.01). Furthermore, harmonious work passion was positively related to job performance (*r* = 0.36, *p* < 0.01), whereas performance pressure is negatively related to job performance (*r* = −0.05, *p* < 0.05). These correlation analysis results provided preliminarily support with the research hypotheses.

**Table 3:** Descriptive statistics and correlations.

**Variables**	**1**	**2**	**3**	**4**	**5**	**6**	**7**	**8**	**9**	**10**
1. Employee gender^a^	—									
2. Employee age^b^	0.06	—								
3. Employee education^c^	−0.10	−0.22^*^	—							
4. Organizational tenure^d^	0.05	0.60^*^	−0.07	—						
5. Employee position level^e^	−0.05	0.23^**^	0.07	0.21^**^	—					
6. Leader perfectionism	−0.02	−0.05	−0.06	−0.11	−0.18^**^	**(0.80)**				
7. Harmonious work passion	0.12	0.11	−0.10	0.02	0.01	0.49^**^	**(0.82)**			
8. Performance pressure	−0.06	−0.06	0.18^*^	0.03	−0.01	0.35	0.36^**^	**(0.77)**		
9. Self-efficacy	−0.05	0.15^*^	0.05	0.13^*^	0.15^*^	0.38^**^	0.58^**^	0.41^**^	**(0.79)**	
10. Job performance	0.01	0.12^*^	0.03	0.03	−0.07	0.03	0.16^**^	−0.05^*^	0.13^*^	**(0.89)**
Mean	0.45	2.55	2.58	2.57	1.35	3.90	3.80	3.79	3.95	4.14
Standard deviation	0.50	1.25	0.73	1.70	0.62	0.57	0.54	0.55	0.48	0.63

*N* = 277. Reliabilities are presented in bold within the parentheses on the diagonal. ^a^Employee gender: male = 0; female = 1. ^b^Employee age: 25 or below = 1; 26–30 = 2; 31–35 =3; 36–40 = 4; 41–45 = 5; 46 or above = 6. ^c^Employee education: high school or below = 1; junior college degree = 2; bachelor's degree = 3; master's degree or above = 4. ^d^Organizational tenure: 2 or below = 1; 2–4 (excluding 2) = 2; 4–6 (excluding 4) = 3; 6–8 (excluding 6) = 4; 8–10 (excluding 8) = 5; 10 or below = 6. ^e^Employee position level: front-line staff = 1; middle employee = 2; senior management = 3. ^*^p < 0.05; ^**^p < 0.01.

### 4.4 Hypothesis testing

Table 4 reports the results of path analysis, and Table 5 reports the findings for the indirect and conditional indirect effects.

**Table 4:** Unstandardized estimates of the mediation path model.

	**Outcome variables**		
	**Harmonious work passion**	**Performance pressure**	**Job performance**
**Control variables**			
Employee gender	0.03 (0.07)	−0.04 (0.06)	−0.04 (0.08)
Employee age	0.03 (0.02)	−0.03 (0.03)	−0.01^*^ (0.04)
Employee education	−0.03 (0.04)	0.09 (0.05)	0.03 (0.06)
Organizational tenure	−0.02 (0.02)	0.04 (0.02)	0.02^*^ (0.03)
Employee position level	0.04 (0.05)	0.06 (0.05)	−0.08 (0.08)
**Independent variable**			
Leader perfectionism	0.45^***^ (0.07)	0.35^***^ (0.06)	−0.03 (0.07)
**Mediators**			
Harmonious work passion			0.26^***^ (0.07)
Performance pressure			−0.17^*^ (0.07)

*N* = 277. ^***^p < 0.001; ^**^p < 0.01; ^*^p < 0.05. Standard error is presented in the parentheses at the diagonal.

**Table 5:** Monte Carlo test results for mediating effects.

**Path**	**Indirect effect (SE)**	**95% CI**	
		**LLCI**	**ULCI**
Path 1: Leader perfectionism → Harmonious work passion → Job performance	0.12 (0.04)	0.05	0.20
Path 2: Leader perfectionism → Performance pressure → Job performance	−0.06 (0.03)	−0.13	−0.01
Path 3: Leader perfectionism → Job performance	−0.03 (0.07)	−0.17	0.11

We proposed that leader perfectionism is positively related to subordinates' harmonious work passion, and that harmonious work passion is positively related to job performance (Hypothesis 1). Our findings showed that leader perfectionism was significantly and positively related to harmonious work passion (β = 0.45, *p* < 0.001), and harmonious work passion significantly and positively related to job performance (β = 0.26, *p* < 0.001). Based on 20,000 bootstrapped samples, the indirect effect of leader perfectionism on employees' job performance through harmonious work passion was 0.12 with 95% confidence interval (CI) of [0.05, 0.20]. Therefore, Hypothesis 1 was supported.

We also proposed that leader perfectionism is positively related to subordinates' performance pressure, and that performance pressure is negatively related to job performance (Hypothesis 2). Leader perfectionism was significantly and positively related to performance pressure (β = 0.35, *p* < 0.001), whereas performance pressure significantly and negatively related to job performance (β = −0.17, *p* < 0.05). The indirect effect of leader perfectionism on employees' job performance through performance pressure was significant and negative (coefficient = −0.06, 95% CI [-0.13, −0.01]), thus providing support for Hypothesis 2.

**Figure 2:**
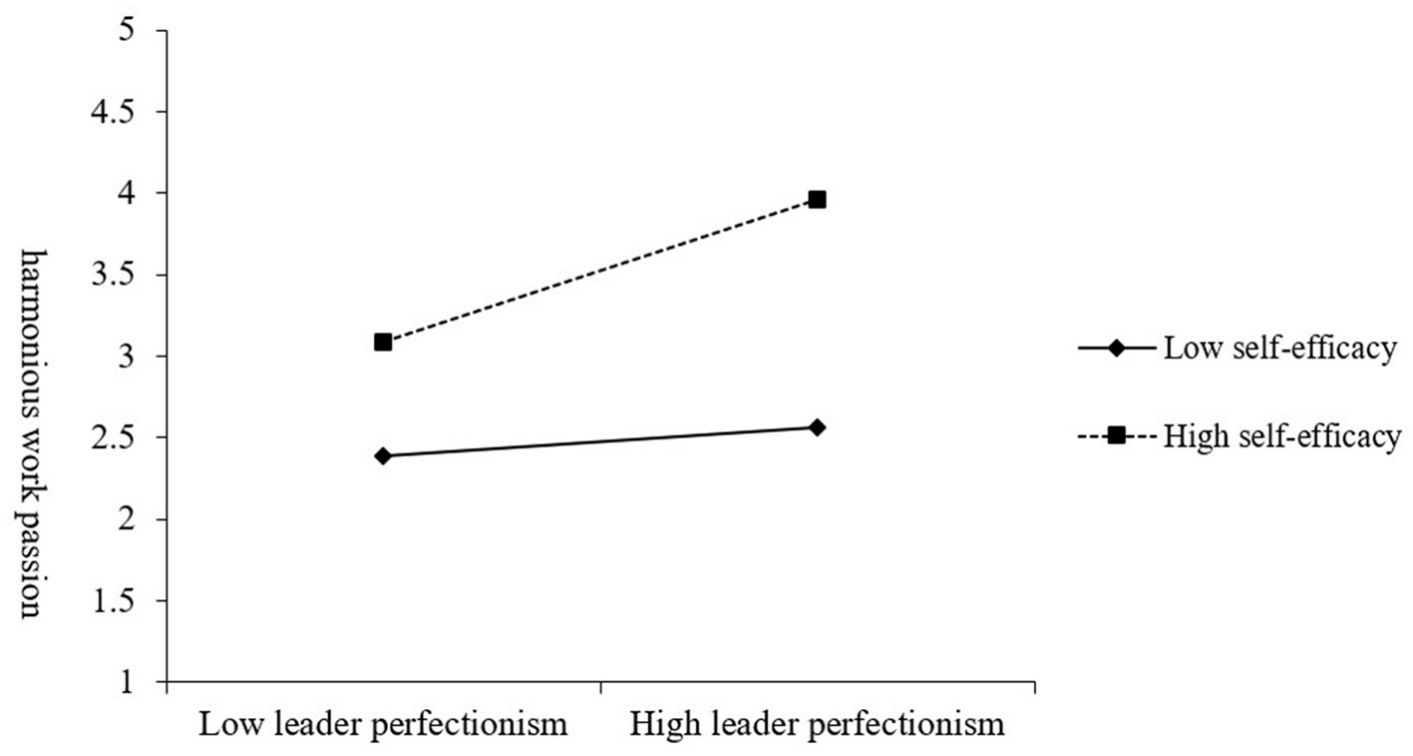
Moderating effect of employee self-efficacy on the relationship between leader perfectionism and harmonious work passion.

**Figure 3:**
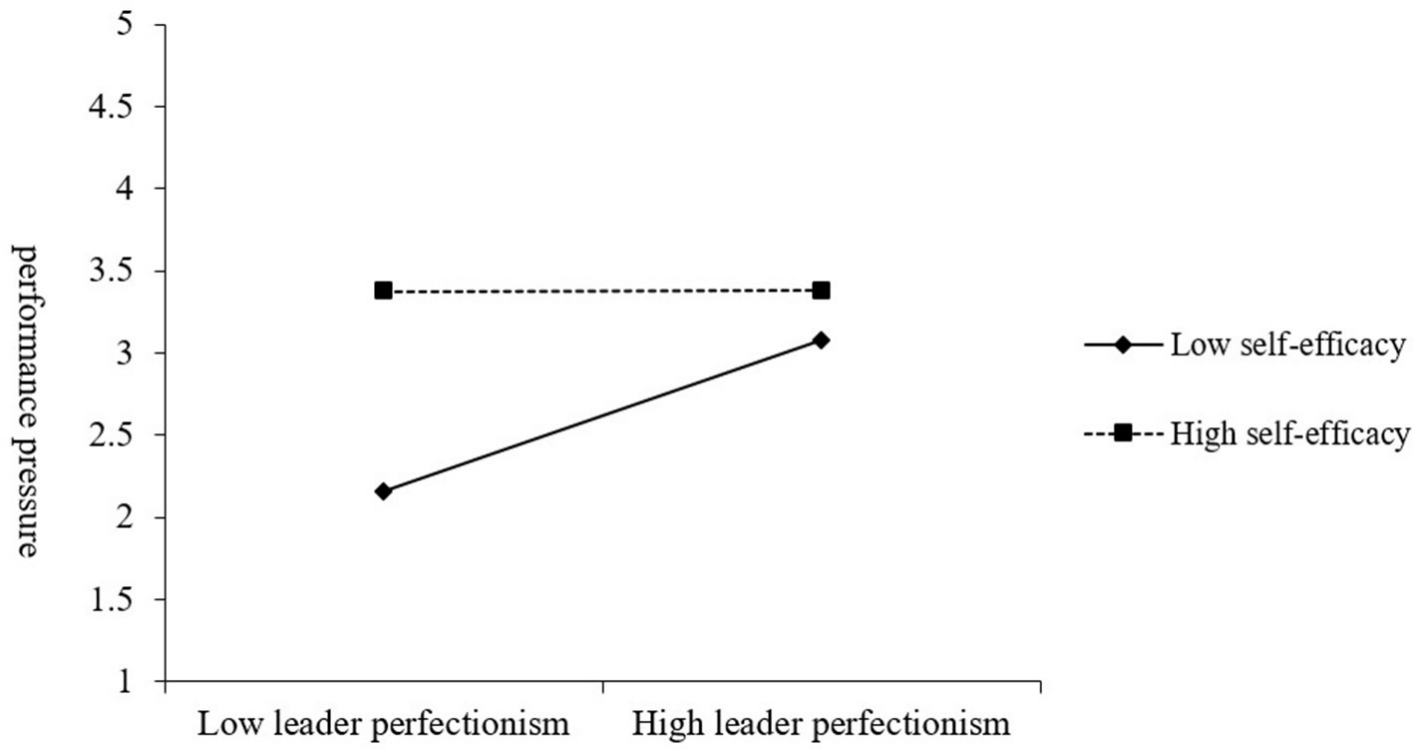
Moderating effect of employee self-efficacy on the relationship between leader perfectionism and performance pressure.

As the results in Table 6 show, the interaction of self-efficacy and leader perfectionism positively related to harmonious work passion (β = 0.17, *p* < 0.05), whereas negatively related to performance pressure (β = −0.23, *p* < 0.05). In Figures 2, 3, we graphed the relationships of leader perfectionism with harmonious work passion and performance pressure respectively, at higher (+1 SD) and lower (−1 SD) levels of self-efficacy. The simple slope analysis indicates that leader perfectionism was positively associated with harmonious work passion at high levels of self-efficacy (simple slope = 0.28, *p* < 0.01), but it exhibited a non-significant association at low levels of self-efficacy (simple slope = 0.06, *n.s*.). Similarly, leader perfectionism was positively associated with performance pressure at high levels of self-efficacy (simple slope = 0.30, *p* < 0.01), but it exhibited a non-significant association at low levels of self-efficacy (simple slope = 0.01, *n.s*.). Therefore, Hypothesis 3a and 3b were supported.

**Table 6:** Path analysis results for moderated moderating effects.

	**Outcome variables**		
	**Harmonious work passion**	**Performance pressure**	**Job performance**
**Control variables**			
Employee gender	0.06 (0.05)	−0.05 (0.06)	−0.04 (0.08)
Employee age	0.02 (0.02)	−0.04 (0.03)	−0.01 (0.04)
Employee education	−0.03 (0.03)	0.13^*^ (0.05)	0.03 (0.06)
Duration of collaboration	−0.04^*^ (0.02)	0.02 (0.02)	0.02 (0.03)
Employee position level	0.01 (0.05)	0.03 (0.04)	−0.08 (0.08)
**Independent variable**			
Leader perfectionism	0.26^***^ (0.06)	0.23^***^ (0.06)	−0.05 (0.07)
**Mediators**			
Harmonious work passion			0.21^***^ (0.07)
Performance pressure			−0.19^*^ (0.08)
**Moderator**			
Self-efficacy	0.52^***^ (0.08)	0.38^***^ (0.07)	
**Interaction effect**			
Leader perfectionism × Self-efficacy	0.17^*^ (0.09)	−0.23^*^ (0.11)	

^***^p < 0.001; ^**^p < 0.01; ^*^p < 0.05. Standard error is presented in the parentheses at the diagonal.

To test the moderated mediating effects, we used the Monte Carlo simulation and tested the indirect effects of leader perfectionism on employees' job performance via harmonious work passion at higher (+1 SD) and lower (−1 SD) levels of self-efficacy and the difference between these two effects. As shown in Table 7, the results show the indirect effect of leader perfectionism on employees' job performance via harmonious work passion is significant when self-efficacy is high (indirect effect = 0.09, 95 % CI [0.03, 0.16]). The difference between these indirect effects is significant (Δ*b* = 0.04, 95 % CI [0.01, 0.10]), thereby supporting Hypothesis 4a. Similarly, as predicted, the results in reveal that the indirect effect of leader perfectionism on employees' job performance via performance pressure is significant when self-efficacy is low (indirect effect = −0.08, 95 % CI [−0.15, −0.02]) but is not significant when self-efficacy is high (indirect effect = −0.03, 95 % CI [−0.07, 0.01]). The difference between these indirect effects is also significant (Δ*b* = 0.05, 95 % CI [0.01, 0.12]). Hence, Hypothesis 4b is supported.

**Table 7:** Test results for moderated mediation effects.

**Path**	**Self-efficacy**	**Indirect effect**	**95% CI**
Leader perfectionism → Harmonious work passion → Job performance	High (+1SD)	0.09	[0.03, 0.16]
	Low (−1SD)	0.05	[0.01, 0.10]
	Difference	0.04	[0.01, 0.10]
Leader perfectionism → Performance pressure → Job performance	High (+1SD)	−0.03	[−0.07, 0.01]
	Low (−1SD)	−0.08	[−0.15, −0.02]
	Difference	0.05	[0.01, 0.12]

## 5 Discussion

Based on appraisal theory of emotion, we investigated the double-edged sword effects of leader perfectionism on employees' job performance. Across the three waves of field study, the results indicated that: (1) Leader perfectionism stimulates positive emotional appraisal in employees, enhancing their harmonious work passion and ultimately improving job performance; (2) The excessively high performance standards associated with leader perfectionism can also trigger negative appraisal, leading to reduced employees' job performance; (3) Employee self-efficacy plays a crucial moderating role in the process of leader perfectionism affecting job performance. Employees with high self-efficacy can better regulate their emotions and, when faced with high performance demands from perfectionistic leaders, can increase their level of harmonious work passion, thereby improving job performance. Conversely, employees with low self-efficacy may feel overwhelmed by leader perfectionism, reducing their job performance.

### 5.1 Theoretical implications

Our study makes several theoretical contributions. First, our study provides a novel explanation for the paradoxical effects of leader perfectionism on followers' work outcome. Previous studies have mostly focused on the negative influence of perfectionism, believing that high expectations, pressure, and distrust of others will lead to a series of maladaptive consequences (e.g., Dunkley et al., 2006; Sherry et al., 2007; Kinman and Grant, 2022), while the bright side of perfectionism has not been studied enough. However, we challenge prevailing conclusions and find that leader perfectionism can be both a boon and a bane for employees' job performance, and that these two opposing mechanisms exist simultaneously. Investigating both the positive and negative effects of perfectionism extend our understanding of how and why these behaviors benefit and harm actors and organizations, deepening our understanding of the contradictory nature of perfectionism. Especially this is true for leaders, who are one of the most influential roles in the workplace (Avolio et al., 2009). Thus, by untangling the mixed effects of leader perfectionism on employees' job performance, we provide a more balanced and dialectical understanding of the effects of leader perfectionism than previously assumed, and highlight the need to consider the potential bright side of leader perfectionism.

Second, our study strengthens the literature link between appraisal theory of emotion and perfectionism. Existing research have analyzed the double-edged sword effect of leader perfectionism on subordinates from the perspective of leader-member relationship and resource perspective (Song et al., 2022; Gunyakti Akdeniz et al., 2024). Appraisal theory of emotion argues that emotions are generated based on the emotional appraisal of events and situations (Lazarus, 1991a). This perspective provides a promising perspective to explain the inconsistent findings of previous studies. In this way, we examine the mediating mechanism of positive and negative coexistence between leader perfectionism and employees' job performance driven by different emotional appraisal. Therefore, our study opens the black box between perfectionism and job performance at the interpersonal level, answering the calls for studies on theory-based mediators (Harari et al., 2018).

Third, our study contributes to the emotional appraisal literature by expanding the positive and negative emotional outcomes caused by leader perfectionism. Based on appraisal theory of emotion, in the face of complex external situations, individuals may have both positive and negative emotions (Roseman, 1991). In this way, we carefully analyze the different emotional paths (i.e., harmonious work passion and performance pressure) of leader perfectionism and employees' job performance. If these two emotional paths are ignored, scholars cannot better understand the performance differences caused by workplace perfectionism.

Finally, our study helps to address this inconsistency by introducing a key boundary condition—self-efficacy. As a common personality characteristic, self-efficacy reflects an individual's belief in ability to perform a particular job task. The results showed that employees with high self-efficacy had more positive emotional appraisal, while those with low self-efficacy had the opposite effect. These findings expand the perfectionism literature by clarifying the boundary conditions for the perfectionism—job performance relationship, emphasizing the importance of considering employee differences in the leader perfectionism area.

### 5.2 Practical implications

The findings of this study offer important managerial implications for practice. First, cultivate employees' self-efficacy. Leaders should pay attention to the cultivation of employees' self-efficacy, that is, employees' confidence and ability to complete specific tasks. By providing the necessary training, feedback, and support, leaders can help employees improve their self-awareness, making them more aware of their strengths and areas for improvement. In addition, leaders should encourage employees to face challenges and overcome difficulties to increase self-confidence and improve work performance.

Second, the rational use of leader perfectionism. Leader perfectionism refers to the leader's high standards and strict requirements for work results. Moderate perfectionism can push employees to pursue excellence and improve the quality of their work. However, excessive perfectionism can cause employees to feel great stress, affecting their mental health and job satisfaction. Therefore, leaders need to balance perfectionist expectations, ensuring that they are both challenging and not overly stressful for employees.

Third, understand the mechanism of performance pressure. Leaders should deeply understand how leader perfectionism affects job performance through performance pressure. This means that leaders need to recognize how their expectations and behaviors translate into employee job stress and how that stress affects employee performance. By understanding this mechanism, leaders can manage their teams more effectively and reduce unnecessary stress while maintaining the pursuit of high quality.

### 5.3 Limitations and future research

Our study revealed the mechanism that leader perfectionism influences job performance through harmonious work passion and performance pressure, and highlighted the critical role of self-efficacy. However, the study has limitations. First, despite using multi-wave and multi-source survey data and testing for common method bias using Harman's single-factor method, there are limitations in testing for causality. Therefore, we encourage future research to use full-cycle method including laboratory design or longitudinal design to improve the accuracy of study results (Chatman and Flynn, 2005). For example, by measuring leader perfectionism at different points in time, researchers can determine whether and how levels of perfectionism change over time, giving us insight into its change effects. Second, this study solely explained the impact mechanism of leader perfectionism on subordinates' job performance from an emotional perspective, but the high goal orientation and excessive work investment feature of perfectionism (Slade and Owens, 1998) might lead to excessive resource depletion in subordinates, triggering resource protection reactions (Hobfoll et al., 2018), also trigger cognitive resource overload and lead to ego depletion of subordinates (Baumeister et al., 1998). For example, leader perfectionism expects subordinates to demonstrate the same perfectionist pursuit and meet their high standards, resulting in lower intrinsic motivation and increased self-criticism. Therefore, future research could analyze the impact mechanisms of leader perfectionism on subordinates' behavior or performance from other theoretical perspectives, such as resource and motivated cognition views.

Third, the sample was primarily from South China, so the findings might be influenced by regional factors. Perfectionism traits can vary across different cultural backgrounds (Smith et al., 2017), and our findings need to be validated in other cultural contexts to verify the generality. The fourth limitation is our study only examined the moderating role of self-efficacy, but other boundary conditions may exist. Future research could delve into boundary conditions like job resources, organizational support, and psychological capital in the relationship between leader perfectionism and employee job performance.

Finally, we focused on other-oriented perfectionism (i.e., leader perfectionism) in our study, which of course cannot depict a full picture of the association workplace perfectionism and behavior at work. For example, our study viewed leader perfectionism as other-oriented perfectionism of the three types of perfectionism, and did not discuss whether and how the other two types of perfectionism have an impact on leader itself or others. Specifically, whether the manager's attitude or behavior toward perfection is transmitted on to supervisors or even subordinates (i.e., the trickle-down effect) (Wo et al., 2019). At the same time, we used a single-dimension scale to measure leader perfectionism. Recent research shows that perfectionism contains at least two dimensions, representing “the setting and striving for excessively high and often unrealistic standards, accompanied by frequent thoughts focused on attainment of these standards and overly critical self-evaluation” (Sirois and Molnar, 2016, p. 1). Therefore, research on perfectionism in the workplace needs to be further expanded. We encourage future research to conceptualize and develop new measurement of perfectionism in the workplace to explore the interpersonal and intrapersonal effects of perfectionism with a more holistic perspective.

## Data Availability

The original contributions presented in the study are included in the article/supplementary material, further inquiries can be directed to the corresponding author.

## Footnotes

^1^Considering the dualistic model of work passion (Vallerand et al., 2003), leader perfectionism's pursuit of performance will form a performance climate and then lead to obsessive work passion (Zhang et al., 2022). However, extant research show no direct or indirect relationship between obsessive work passion and performance (e.g., Ho et al., 2011; Astakhova and porter, 2015; Pollack et al., 2020), implying that obsessive work passion may not be the mediator of the negative pathway between leader perfectionism and employees' job performance. In view of great attention of performance from leader perfectionism (Guo et al., 2020), we suppose performance pressure – rather than obsessive work passion - serve as the mediator for negative pathway.
